# Amylases StAmy23, StBAM1 and StBAM9 regulate cold-induced sweetening of potato tubers in distinct ways

**DOI:** 10.1093/jxb/erx076

**Published:** 2017-03-28

**Authors:** Juan Hou, Huiling Zhang, Jun Liu, Stephen Reid, Tengfei Liu, Shijing Xu, Zhendong Tian, Uwe Sonnewald, Botao Song, Conghua Xie

**Affiliations:** 1 Key Laboratory of Potato Biology and Biotechnology, Ministry of Agriculture, Wuhan 430070, People’s Republic of China; 2 National Center for Vegetable Improvement (Central China), Wuhan 430070, People’s Republic of China; 3 Huazhong Agricultural University, Wuhan 430070, People’s Republic of China; 4 Key Laboratory of Horticultural Plant Biology (HZAU), Ministry of Education, Wuhan 430070, People’s Republic of China; 5 College of Forestry, Henan University of Science and Technology, Luoyang 471003, People’s Republic of China; 6 Biochemistry Division, Department of Biology, Friedrich-Alexander-University Erlangen-Nuernberg, 91058 Erlangen, Germany

**Keywords:** α-Amylase, β-amylase, cold-induced sweetening, potato, reducing sugar, starch degradation, tuber.

## Abstract

Cold-induced sweetening (CIS) in potato is detrimental to the quality of processed products. Conversion of starch to reducing sugars (RS) by amylases is considered one of the main pathways in CIS but is not well studied. The amylase genes *StAmy23*, *StBAM1*, and *StBAM9* were studied for their functions in potato CIS. StAmy23 is localized in the cytoplasm, whereas StBAM1 and StBAM9 are targeted to the plastid stroma and starch granules, respectively. Genetic transformation of these amylases in potatoes by RNA interference showed that β-amylase activity could be decreased in cold-stored tubers by silencing of *StBAM1* and collective silencing of *StBAM1* and *StBAM9*. However, *StBAM9* silencing did not decrease β-amylase activity. Silencing *StBAM1* and *StBAM9* caused starch accumulation and lower RS, which was more evident in simultaneously silenced lines, suggesting functional redundancy. Soluble starch content increased in RNAi-*StBAM1* lines but decreased in RNAi-*StBAM9* lines, suggesting that StBAM1 may regulate CIS by hydrolysing soluble starch and StBAM9 by directly acting on starch granules. Moreover, StBAM9 interacted with StBAM1 on the starch granules. *StAmy23* silencing resulted in higher phytoglycogen and lower RS accumulation in cold-stored tubers, implying that StAmy23 regulates CIS by degrading cytosolic phytoglycogen. Our findings suggest that StAmy23, StBAM1, and StBAM9 function in potato CIS with varying levels of impact.

## Highlight

StAmy23, StBAM1 and StBAM9 play distinct roles in potato cold-induced sweetening by preferentially acting on soluble phytoglycogen, soluble starch and starch granules, respectively, in different subcellular locations.

## Introduction

Potato (*Solanum tuberosum* L.) is the most important non-grain food crop in the world. Significant amounts of potatoes are used for making crisps, French fries and other products. For a continuous supply of raw material, potato tubers are often stored at low temperature to reduce sprouting, water loss and pathogenesis. However, cold storage (normally less than 10 °C) often leads to accumulation of reducing sugar (RS) in tubers, which is known as cold-induced sweetening (CIS). RS reacts with α-amino acid groups of nitrogenous compounds during frying resulting in a Maillard browning of the products accompanied by harmful acrylamide accumulation (Tareke *et al.*, 2002; Shepherd *et al.*, 2010). Acrylamide formation correlated well with the concentrations of RS (Amrein *et al.*, 2003), and there was a positive correlation between acrylamide content of potato crisps and colour (Pedreschi *et al.*, 2005)—the higher the acrylamide accumulated, the darker the crisp colour will be. Therefore, CIS poses a significant challenge to the potato industry and raises a worldwide food safety concern (Xin and Browse, 2000; Mottram *et al.*, 2002; Halford *et al.*, 2012).

Sucrose hydrolysis by invertase and starch degradation have been reported to be the main pathways involved in potato CIS (Blenkinsop *et al.*, 2003; Bhaskar *et al.*, 2010; Zhang *et al.*, 2014*b*; Lin *et al.*, 2015). The invertase activity is considered critical for sucrose cleavage (Bhaskar *et al.*, 2010), and a protein complex, StvacINV1–StInvInh2B–SbSnRK1, is implicated in the regulation of the enzyme activity in cold-stored tubers (Lin *et al.*, 2015). Nevertheless, specific amylase genes responsible for potato CIS remain to be elucidated. Starch degradation is either hydrolytic (via amylases) or phosphorolytic (via starch phosphorylases). In both cases, the semi-crystalline structures of starch granules must be solubilized. Reversible glucan phosphorylation at the granule surface is essential for complete starch breakdown (Silver *et al.*, 2014). The semi-crystalline matrix of the starch granule surface is disrupted by phosphorylation mediated by glucan, water dikinase (GWD) and phosphoglucan, water dikinase (Baunsgaard *et al.*, 2005; Kötting *et al.*, 2005; Xu *et al.*, 2016). In potato leaves, the phosphorylation level of the starch granule surface is increased during starch breakdown (Ritte *et al.*, 2004). In potato tubers, approximately 0.5% of the glucose residues are phosphorylated, which is considered highly phosphorylated (Mikkelsen *et al.*, 2006). The analysis of transgenic potato plants with decreased GWD expression revealed that starch degradation was largely reduced in cold stored potato tubers, which was accompanied by a reduction in RS accumulation (Lorberth *et al.*, 1998).

The hydrolytic pathway of starch degradation involves α-amylase (AMY) and β-amylase (BAM). AMY is an endoamylolytic enzyme that specifically hydrolyses α-1,4-glucan bonds to yield various linear and branched malto-oligosaccharides. Multiple genes encode different amylase isoforms that may have different roles depending on plant tissues and species. For example, suppressing rice α-amylase I-1 resulted in increased starch accumulation in young leaves under a sugar-supplemented condition (Asatsuma *et al.*, 2005; Kitajima *et al.*, 2009). In contrast, in Arabidopsis, all *AtAMY* single, double and triple knockout mutants displayed normal starch breakdown (Yu *et al.*, 2005; Glaring *et al.*, 2011). A recent study revealed that two α-amylase genes (*StAmy1* and *StAmy23*) were expressed in potato tubers, but only *StAmy23* transcripts could be induced by low temperature (Zhang *et al.*, 2014*a*). The cold-responsive nature of this amylase gene was also reported in apple, where the expression of *Amy8*, which shows the highest identity to potato *Amy23*, was transiently upregulated at 0.5 °C in fruit (Wegrzyn *et al.*, 2000). These observations imply that Amy23 may function under low-temperature conditions.

BAM belongs to the glycosyl hydrolase 14 family and hydrolyses α-1,4-linked glucan chains from the non-reducing end and catalyses the release of β-maltose (Weise *et al.*, 2005). It is believed that the major pathway of starch degradation occurs via BAMs in Arabidopsis and other organisms (Scheidig *et al.*, 2002; Fulton *et al.*, 2008; Valerio *et al.*, 2011; Monroe *et al.*, 2014). The Arabidopsis genome codes for nine BAMs with varying functions in starch hydrolysis (Fulton *et al.*, 2008; Li *et al.*, 2009; Reinhold *et al.*, 2011; Valerio *et al.*, 2011; Monroe *et al.*, 2014; Soyk *et al.*, 2014). Some isoforms of BAM are considered cold-responsive. BAM3 contributes to leaf starch degradation in mesophyll cells at night and under cold stress (Kaplan and Guy, 2005; Fulton *et al.*, 2008; Monroe *et al.*, 2014). RNAi-*BMY8* lines of Arabidopsis exhibited less maltose accumulation in response to cold stress (Kaplan and Guy, 2004, 2005). Cold-treated *bam5/1* plants revealed an increase in *BAM3* transcripts and reducing sugars (Monroe *et al.*, 2014). Similar results were reported from other species. Overexpression of *PtrBAM1* from *Poncirus trifoliata* in tobacco caused an increase in BAM activity and starch degradation, which was accompanied by a greater accumulation of maltose and soluble sugars at room temperature or under cold stress (Peng *et al.*, 2014). The β-amylase activity and reducing sugar content increased sharply in five potato cultivars during the first week of storage at 4 °C (Cottrell *et al.*, 1993). When the storage temperature was reduced from 20 °C to 5 or 3 °C, the β-amylase activity of potato tubers was enhanced by 4- to 5-fold over a 10-day period, together with maltose accumulation (Nielsen *et al.*, 1997). Recent research demonstrated a substantial increase in β-amylase expression and an abundant accumulation of reducing sugars in potato tubers cooled to 3–5 °C (Wiberley-Bradford *et al.*, 2016). These results suggest that β-amylase may play a significant role in potato CIS.

Seven BAMs (StBAM1, StBAM3, StBAM4, StBAM5, StBAM7, StBAM8, and StBAM9) were identified from the potato genome. Moreover, *StBAM1* and *StBAM9* showed higher transcripts in tubers than the others and were strongly induced by low temperature (Zhang *et al.*, 2014*a*). Tomato Affymetrix GeneChip analysis showed significant up-regulation of *StBMY7* and *PCT-BMY1* in potato tubers exposed to low temperature (Bagnaresi *et al.*, 2008). By modulating the expression of amylase inhibitor gene *SbAI* through potato transformation, the obvious changes in β-amylase activity resulted in a decrease in RS accumulation in cold-stored over-expressing tubers, and protein–protein interactions occurred between SbAI and StAmy23, and StBAM1 and StBAM9 (Zhang *et al.*, 2014*b*). Thus, it is important to study the functional mechanism of the CIS-associated amylase genes for a systematic dissection and efficient control of potato cold-induced sweetening.

Here, we clarified the phylogeny of the α-amylase and β-amylase gene family in plants and provide the first report on the locations of α-amylase StAmy23 and β-amylases StBAM1 and StBAM9 in potato, together with the interaction between StBAM1 and StBAM9. Moreover, their roles in starch degradation and potato CIS were elucidated by individual gene silencing (*StAmy23*, *StBAM1*, and *StBAM9*) and collective silencing of both *StBAM1* and *StBAM9* in CIS-sensitive potato genotype and metabolomics analysis of the transgenic tubers.

## Materials and methods

### Phylogenetic analysis and alignment of plant amylases

The amylase sequences from different species were obtained from public databases (Supplementary Table S1 at *JXB* online). Phylogenetic analysis of glucosyl hydrolase domains of β-amylase was performed using Phylogeny.fr (Dereeper *et al.*, 2008), and the phylogram was displayed with iTOL online software (Letunic and Bork, 2016). Exon–intron structures were analysed using the Gene Structure Display Server (http://gsds.cbi.pku.edu.cn/). Phylogenetic analysis of α-amylase was performed by MUSCLE. Multiple alignments of the glucosyl hydrolase domains of β-amylase were performed using the ClustalX program with default settings and displayed by Jalview (http://www.jalview.org/). The crystal structure of the soybean β-amylase GmBMY1 (PDB ID 1BYB) was used for alignment analysis.

### Gene cloning and subcellular localization

Based on the Potato Genome Sequencing Consortium (PGSC) database, *StAmy23*, *StBAM1*, and *StBAM9* were cloned from CIS-sensitive cultivar E-potato 3 (E3) cDNA with specific primers shown in Supplementary Table S2. To analyse the locations of StAmy23, StBAM1, and StBAM9, the N-terminal regions of three proteins were analysed for the presence of possible chloroplast transit peptides or signal peptides using ChloroP (http://www.cbs.dtu.dk/services/ChloroP/), TargetP (http://www.cbs.dtu.dk/services/TargetP/) and iPSORT (http://ipsort.hgc.jp/index.html). The open reading frames (ORFs) of these genes without a termination codon were amplified with specific primers modified to contain the Gateway (Invitrogen) attB recombination sites. PCR products were purified and recombined into pDONR221 (Invitrogen) to generate entry clones via BP reactions. C-terminal green fluorescent protein (GFP) fusions of StAmy23–GFP, StBAM1–GFP and StBAM9–GFP were performed by recombining the entry clones with pK7FWG2 driven by the 35S silencing promoter using LR clonase (Invitrogen). For a starch granule marker, full-length granule-bound starch synthase I (StGBSS) without a stop codon was amplified from E3 cDNA with specific primers and cloned into pJCV55 obtained by restriction with *Bgl*II using Exnase II (Vazyme). Primer sequences are shown in Supplementary Table S2. *Agrobacterium tumefaciens* (GV3101) containing StAmy23–GFP, StBAM1–GFP, StBAM9–GFP and StGBSS–red fluorescent protein (RFP) were pressure infiltrated into the leaves of 4-week-old *Nicotiana benthamiana* plants*. A. tumefaciens* was resuspended in agroinfiltration medium at a final concentration of OD_600_=0.1. For coexpression, *Agrobacterium* cultures carrying the appropriate vectors were mixed before infiltration. Two days after infiltration, cells expressing fluorescent protein fusions were observed using a Carl Zeiss AXIO Observer A1 inverted fluorescence microscope.

### Western blotting

Leaf discs (200 mg) expressing StAmy23–GFP, StBAM1–GFP, StBAM9–GFP, StGBSS–RFP, GFP and RFP were harvested at 2.5 dpi, ground in liquid nitrogen, suspended in 800 μl of protein extraction buffer (25 mM Tris–HCl pH 7.5, 1 mM EDTA, 150 mM NaCl, 10% glycerol, 10 mM DTT, 2% PVPP, 1% (v/v) protease inhibitor cocktail (Sigma-Aldrich; P0044), 0.1% Tween-20) on ice for 30 min, and centrifuged for 15 min at 2000 *g* at 4 °C. The supernatant was incubated by addition of 5×SDS-PAGE loading buffer, heated to 99 °C for 10 min, and analysed by 10% SDS-PAGE. Following blotting with blocking buffer (5% skimmed milk in phosphate buffered saline with Tween 20), hybridization was performed using GFP antibody (anti-GFP pAb-HRP-DirecT) (MBL; 598-7) and RFP antibody (anti-RFP pAb-HRP-DirecT) (MBL; PM005-7) diluted by the hybridization buffer (1% skimmed milk in phosphate-buffered saline) according to the manufacturer’s protocols.

### Vector construction and potato transformation

To construct the *StBAM1* and *StBAM9* RNA interference vector, a 373-bp fragment from 1254 bp downstream of the start codon was amplified from the *StBAM1* cDNA with specific primers (Supplementary Table S2, StBAM1-C1254 and StBAM1-C1626). A 281-bp fragment containing the ORF and the 3′-untranslated region was obtained from *StBAM9* cDNA with specific primers (Supplementary Table S2, StBAM9-T1620 and StBAM9-T1900). The corresponding fragment was subcloned into the entry vector pDONR221 and cloned into the pHellsGate8 vector using Gateway technology (Invitrogen). To construct the RNAi vector of simultaneous repression of both *StBAM1* and *StBAM9*, a 239-bp fragment starting from 751 bp downstream of the *StBAM1* start codon was amplified with primers [Supplementary Table S2, (StBAM1+StBAM9)-1-C751 and (StBAM1+StBAM9)-1-C989], a 218-bp fragment from 976 bp downstream of *StBAM9* start codon was amplified with primers [Supplementary Table S2, (StBAM1+StBAM9)-9-C976 and (StBAM1+StBAM9)-9-C1193]. The amplified PCR products were mixed and treated as templates, a chimeric fragment was obtained by overlap PCR with primers [Supplementary Table S2, (StBAM1+StBAM9)-1-C751 and (StBAM1+StBAM9)-9-C1193] and cloned into the pHellsGate8 vector. Primer sequences are shown in Supplementary Table S2. These constructs were driven by the 35S CaMV promoter, introduced into *A. tumefaciens* strain LBA4404 and transformed into the CIS-sensitive potato cv E3 as previously described (Si *et al.*, 2003). Moreover, plants with RNA interference of *StAmy23* in *S. tuberosum* L. cv. Solara have been previously obtained (Ferreira, 2011).

### Plant material and sampling

The plants were grown at 18–25 °C in 24 cm-diameter plastic pots in the greenhouse (light intensity ranged from 400 to 1000 µmol m^–2^ s^–1^) at the National Centre for Vegetable Improvement (Central China), Huazhong Agricultural University (Wuhan, China). The morphology of plants was observed 8 weeks after planting. When the leaves had senesced naturally, the mature tubers were harvested, and the tuber yield per plant and starch granule size were determined. Harvested tubers were treated and sampled as described by Lin *et al.* (2015).

### RNA isolation and quantitative RT-PCR

The frozen tuber and leaf samples were ground to a fine powder in liquid nitrogen for RNA isolation as described previously (Liu *et al.*, 2013). Quantitative RT-PCR was performed with the Bio-Rad CFX Connect^TM^ Real-Time System (Bio-Rad, USA). Potato gene *ef1a* was used as a reference (Nicot *et al.*, 2005), and the primers for amylase family genes were used as described before (Zhang *et al.*, 2014*a*). Gene expression levels were calculated by the 2^–∆∆Cq^ method as described by Bio-Rad (http://www.bio-rad.com/zh-cn/applications-technologies/real-time-pcr-experimental-design).

### Determination of enzyme activity, starch, soluble starch, sugar, and crisp colour

For amylase extraction, 1 ml of 0.1 M citric acid buffer, pH 5.6, was added to 100 mg of powdered tuber tissue and extracted for 20 min on ice. The extract was centrifuged for 10 min at 4 °C and 2000 *g*, and the amylase activity in the supernatant was determined using assay kits from Megazyme (Bray, Ireland) as previously described (Zhang *et al.*, 2014*b*). Protein quantification in the extraction was performed with a bicinchoninic acid kit (PPLYGEN; P1511). Starch, glucose, fructose and sucrose contents were determined as described previously (Müller-Röber *et al.*, 1992). The soluble starch content was determined by measuring the amount of glucose released by treatment with amyloglucosidase (Smith and Zeeman, 2006). The fry test was carried out, and crisp colour was determined according to Liu *et al.* (2011) with modifications. Briefly, each sampled tuber was peeled and cut in half longitudinally. One part was used for the fry test; the tubers were cut into about 1 mm slices and fried at 170 °C for 3 min or until the cessation of bubbles in a Frymaster (USA) H14 electric fryer. The crisp colour was visually determined by using the Color Standards Reference Chart for Potato Chips from scale 1 (light) to 10 (dark) (Snack Food Association, USA). The crisp colour index (CCI) was calculated for each line with 15 crisps from three tubers by applying the colour scale value (*C*) to the formula CCI = [∑(*C*_*i*_×*N*_*i*_)]/*N*, where *C*_*i*_ is colour scale *i*, *N*_*i*_ is number of crisps of *C*_*i*_ and *N* is the total number of the crisps tested. For the statistical analyses, biological triplicates were used for each measurement, and all the data are presented as means±SD. Significance was determined by Student’s *t* test and LSD test with the software SPSS Statistics v. 20.0 for Windows.

### Bimolecular fluorescence complementation

The ORFs of *StBAM1* and *StBAM9* without termination codon were amplified with specific primers (see Supplementary Table S2) and were separately cloned into NYFP and CYFP vector by restriction with *Bam*HI and *Sal*I using Exnase II (Vazyme). Transient expression by agroinfiltration and fluorescence detection were performed as described above (Gene cloning and subcellular localization).

### Yeast two-hybrid assays

For vector construction, the full-length *StBAM1*, *StBAM9*, *StGBSS*, *StGWD*, *StLSF1 StLSF2*, and *StBAM9* without transit peptides were amplified with specific primers (see Supplementary Table S2) and separately cloned into a pGBKT7 vector by restriction with *Eco*RI and *Sal*I using Exnase II (Vazyme). The vectors pGADT7-StAmy23, pGADT7-StBAM1 and pGADT7-StBAM9 were obtained previously (Zhang *et al.*, 2014*b*). Afterwards, pairwise vectors between pGADT7-StAmy23, pGADT7-StBAM1, pGADT7-StBAM9 and pGBKT7-StBAM1, pGBKT7-StBAM9, pGBKT7-StGBSS, pGBKT7-StGWD, pGBKT7-StLSF1, and pGBKT7-StLSF2 were transformed into yeast strain AH109 with BD Matchmaker Screening Kit according to the manufacturer’s protocols. A positive control was used as previously described (Lin *et al.*, 2013).

## Results

### Multiple genes encode different amylase isoforms in potato

Together with Gene ID, alternative name, chloroplast transit peptide or signal peptide information and confirmed localization, seven β-amylases (StBAM1, StBAM3, StBAM4, StBAM5, StBAM7, StBAM8, and StBAM9) and two α-amylases (StAmy1 and StAmy23) were identified in the potato genome sequence consortium database (http://solanaceae.plantbiology.msu.edu/pgsc_download.shtml) and listed in Table 1.

**Table 1.** General information for potato α-amylases and β-amylases

Gene name	PGSC name^*a*^	Alternative name	No. of amino acids	ChloroP/TargetP	TargetP	Demonstrated localization
				(chloroplast transit peptide)	(signal peptide)	
*StBAM1*	PGSC0003DMG400001549	*StBAM1* ^*b*^	579	Yes/Yes		Plastid stroma (this work)
*StBAM3*	PGSC0003DMG402020509	*StBAM2* ^*b*^, *PCT-BMY1*^*c*^	541	No/No	No	Chloroplast stroma^*c*^
*StBAM4*	PGSC0003DMG400012129	*StBAM3* ^*b*^	541	Yes/Yes		
*StBAM5*	PGSC0003DMG400026199	*StBAM4* ^*b*^	587	Yes/Yes		
*StBAM7*	PGSC0003DMG400000169	*StBAM5* ^*b*^	554	No/No	No	
*StBAM8*	PGSC0003DMG400024145	*StBAM6* ^*b*^	856	No/Yes		
*StBAM9*	PGSC0003DMG400010664	*StBAM7* ^*b*^	535	Yes/Yes		Starch granule (this work)
*StAmy1*	PGSC0003DMG400020603		441	No/No	Yes	
*StAmy23*	PGSC0003DMG400009891		407	No/No	No	Cytoplasm (this work)

^*a*^ PGSC names are from the Potato Genome Sequence Consortium database.

^*b*^ Alternative names are from Zhang *et al.* (2014*a*).

^*c*^ Alternative name and demonstrated localization are from Scheidig *et al.* (2002).

**Fig. 1.**
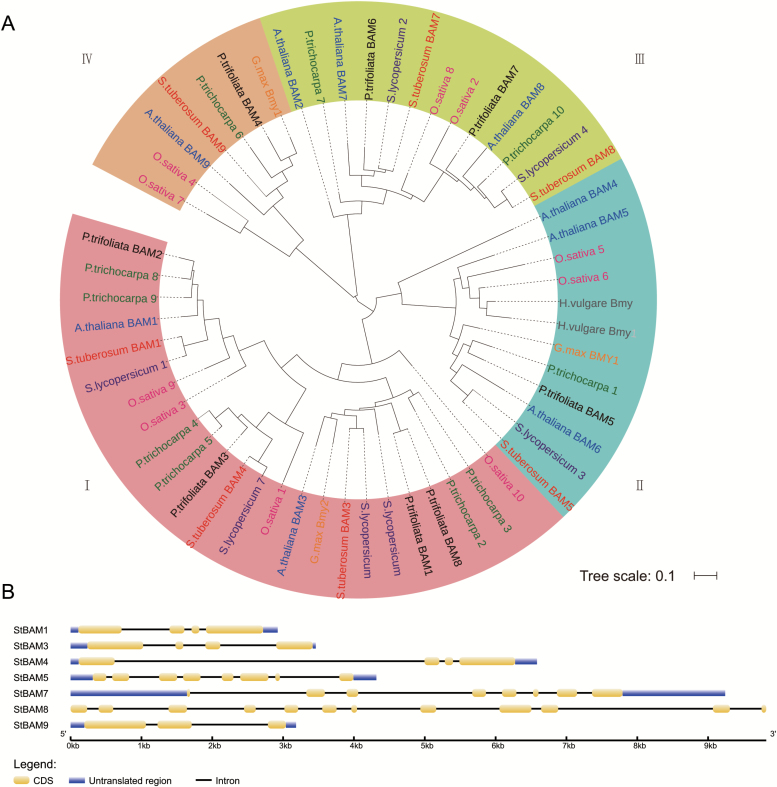
Phylogenetic analysis of β-amylases and gene structures of *StBAM1*–*9*. (A) A phylogram of 56 β-amylases from eight plant species. The glucosyl hydrolase domains of 56 β-amylases were aligned by MUSCLE and were used to construct a maximum-likelihood tree. The robustness of the tree is derived from 100 bootstrap replicates. The scale bar refers to 0.1 amino acid substitution per site. The phylogram displayed with Tree Of Life software (Letunic and Bork, 2016) showed that plant β-amylases were clustered into four subfamilies. Potato proteins are shown in red, tomato proteins in purple, Arabidopsis proteins in blue, rice proteins in rose red, *P. trifoliate* proteins in black, poplar proteins in dark green, soybean proteins in orange, and barley proteins in grey. (B) Gene structures for potato *StBAM*s based on the number and position of CDS (yellow), intron (solid lines) and untranslated region (blue).

A phylogenetic analysis was performed using the conserved glucosyl hydrolase domains of 56 β-amylase proteins from potato (*Solanum tuberosum*), tomato (*Solanum lycopersicum*), Arabidopsis (*Arabidopsis thaliana*), rice (*Oryza sativa*), soybean (*Glycine max*), barley (*Hordeum vulgare*), trifoliate orange (*Poncirus trifoliate*), and poplar (*Populus trichocarpa*) (see Supplementary Table S1), suggesting that β-amylases may be evolutionarily conserved among different species. The analysis displayed four major subfamilies (Fig. 1A). Potato β-amylases StBAM1, StBAM3, and StBAM4 were assigned to subfamily I, StBAM5 to subfamily II, StBAM7 and StBAM8 to subfamily III, while StBAM9 was clustered in subfamily IV. In most cases, the β-amylases, such as BAM1, BAM3, BAM4, BAM5, BAM7, and BAM8 from Solanaceae (potato and tomato) were similar. However, no β-amylase from subfamily IV was found in tomato. Moreover, the intron/exon structures of the *StBAM* gene family were consistent with the phylogenetic analysis. *StBAM*s in the same subfamily possessed similar gene structures except for subfamily III, in which *StBAM7* and *StBAM8* differed in the structure of exons at the 5′ and 3′ ends (Fig. 1B). The structure of *StBAM9*, which had only three exons, was different from other *StBAM* genes, suggesting a distinct function.

**Fig. 2.**
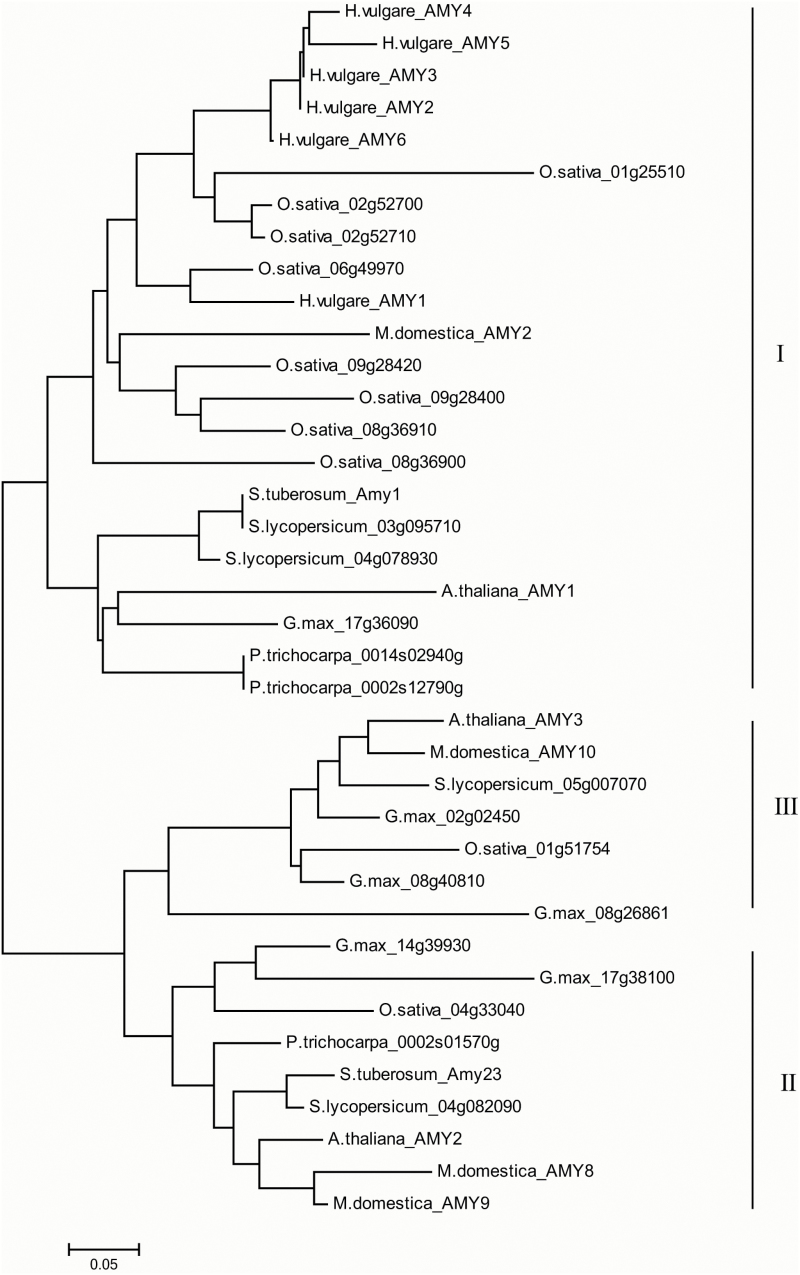
The phylogenetic relations of 38 α-amylases from eight plant species. Thirty-eight α-amylases were aligned by MUSCLE with default settings and used to generate a neighbour-joining tree. The scale bar refers to amino acid substitution per site. The phylogram showed that plant α-amylases were clustered into three subfamilies. α-Amylases of tomato, rice, poplar, and soybean were named in terms of the corresponding locus names, and the names of α-amylase for potato, Arabidopsis, barley, and apple were derived from the reports.

We also performed a phylogenetic analysis of 38 plant α-amylases from potato, tomato, Arabidopsis, rice, soybean, barley, poplar, and apple (see Supplementary Table S1). As shown in Fig. 2, the AMYs were classified into three major subfamilies, represented by AtAMY1, AtAMY2, and AtAMY3. StAmy1 was clustered into subfamily I, which was obviously divided into monocot and dicot proteins except for MdAMY2 from apple. Analysis using iPSORT indicated that StAmy1 possessed a signal peptide (Table 1) and may be targeted to a secretory pathway by its function. StAmy23 belonged to the subfamily II and was most closely related to AtAMY2 from Arabidopsis.

### The structure of StBAM9 predicts an inactive protein

**Fig. 3.**
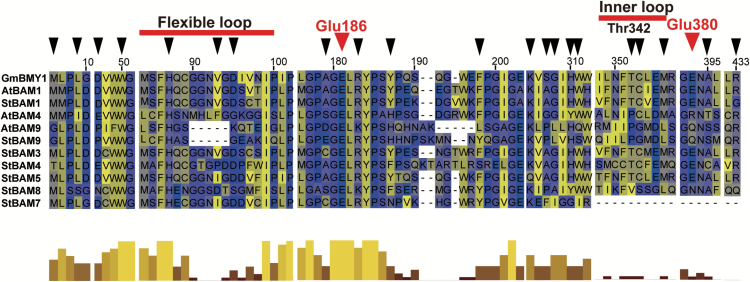
Alignment of the conserved glucosyl hydrolase domains from potato StBAMs, soybean GmBMY1, and Arabidopsis AtBAM1, AtBAM4, and AtBAM9. Most and same colour shading indicates identical residues and conservative substitutions for each column, whereas the less conserved residues are presented as a different colour. An overall picture of sequence conservation is displayed in the bar graph, together with tall yellow bars representing high sequence conservation and short brown bars representing low sequence conservation. Substrate binding sites and two catalytic residues are marked with black and larger red arrowheads, respectively. The deletion and substitution of the flexible loop and inner loop in StBAM9, AtBAM9, and AtBAM4 were indicated with red lines.

The conserved glucosyl hydrolase domains of the potato StBAM proteins, together with Arabidopsis AtBAM1, AtBAM4, and AtBAM9, were aligned with that of the soybean β-amylase GmBMY1 for which the crystal structure information was available (Mikami *et al.*, 1994) (Fig. 3). Based on the structure of GmBMY1, 21 substrate binding residues (black arrowheads) and two active sites (red arrowheads) were identified. Most StBAMs had high sequence similarity in subfamilies I and II, but subfamilies III and IV showed remarkable variations in the flexible outer loop, inner loop and conserved residues. It was noticeable for StBAM9 that its flexible outer loop sequence had five amino acid deletions. The inner loop residue Thr-342 was changed to Pro, and the catalytic residue Glu-380 was replaced with Gln. The results also showed that StBAM9 was similar to AtBAM9 and AtBAM4 in the lack of conserved residues necessary for catalysis (Fulton *et al.*, 2008). It was suggested that the movement of the flexible outer and inner loop affected substrate binding, and the two conserved Glu residues (Glu-186 and Glu-380) controlled catalysis (Mikami *et al.*, 1994). Thus, the structure of StBAM9 suggests that it could be an inactive β-amylase.

### StAmy23, StBAM1 and StBAM9 are localized in the cytoplasm, plastid stroma and starch granules, respectively

We analysed the N-terminal regions of the encoded proteins for the presence of possible chloroplast transit peptides and signal peptides using ChloroP, TargetP, and iPSORT. Both, StBAM1 and StBAM9 possessed predicted chloroplast transit peptides, whereas StAmy23 had no targeting peptide (Table 1).

To discover whether these were chloroplast proteins, the coding regions of StAmy23, StBAM1, and StBAM9 were fused to a sequence encoding an enhanced green fluorescent protein (GFP). The constructs were then expressed transiently by infiltration of transformed *Agrobacterium tumefaciens* cells into *Nicotiana benthamiana* leaves. The construct encoding red fluorescent protein (RFP)-tagged granule-bound starch synthase I (StGBSS–RFP) was used as a positive control for starch granule localization (Szydlowski *et al.*, 2009; Bahaji *et al.*, 2011; Wang *et al.*, 2013), while free GFP and RFP were used for cytosolic localization (Fulton *et al.*, 2008). As expected, free GFP and RFP fluorescence could be observed in the cytosol and nuclei (see Supplementary Fig. S1A, B). Moreover, StGBSS–RFP was mainly localized to starch granules (restricted fluorescence on spherical or oval-shaped structures) rather than in the chloroplast stroma (diffuse fluorescence in the total chloroplast volume) (Supplementary Fig. S1C).

**Fig. 4.**
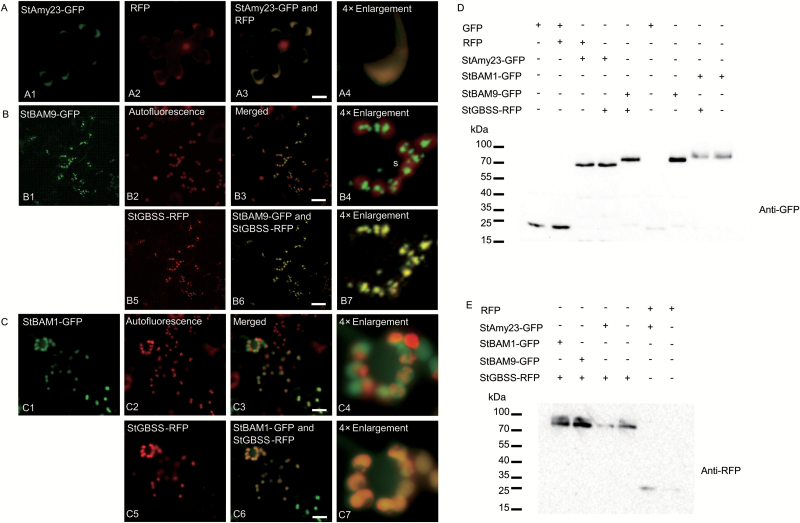
Subcellular localizations of StAmy23, StBAM1, and StBAM9 in *Nicotiana benthamiana* leaves. (A) StAmy23–GFP was coexpressed with cytosol marker RFP (A1–A4). (B) StBAM9–GFP was coexpressed with starch granule marker StGBSS–RFP (B1–B7). (C) StBAM1–GFP was coexpressed with StGBSS–RFP (C1–C7). The first column indicates GFP fluorescence (A1, B1, and C1), the second column indicates RFP fluorescence (A2, B5, and C5) or chlorophyll autofluorescence (B2 and C2), the third column shows merged images, and the last column shows ×4 enlargement of merged images (A4, B4, B7, C4 and C7). Bars: 10 μm. (D, E) Western blots probed with GFP antibody (E) and RFP antibody (F) showing stable protein fusions of potato StAmy23–GFP, StBAM1–GFP, StBAM9–GFP, StGBSS–RFP, and free GFP and RFP with expected size.

As shown in Fig. 4A, the fluorescence from StAmy23–GFP was completely co-localized with the cytosolic regions of free RFP (Fig. 4A1–4), suggesting that StAmy23 was exclusively located in the cytoplasm. In contrast, the non-uniform fluorescence from StBAM9–GFP (Fig. 4B1–4) and StBAM1–GFP (Fig. 4C1–4) coincided with the chlorophyll autofluorescence in most cases, indicating chloroplastic localization. However, the non-uniform distribution and restricted fluorescence rather than diffused fluorescence of StBAM9–GFP within the chloroplasts indicated that StBAM9 might be located on the surface of starch granules in the chloroplasts (Fig. 4B1–4). This speculation was confirmed by coexpression with the starch granule marker StGBSS–RFP. StBAM9–GFP showed full co-localization with StGBSS–RFP indicating that StBAM9 was localized to starch granules (Fig. 4B1, B5–7). Contrastingly, the diffuse fluorescence in the total chloroplast volume for StBAM1–GFP suggested that StBAM1 might be localized mainly in the chloroplast stroma rather than starch granules (Fig. 4C1–4). Interestingly, StBAM1–GFP could be co-localized with the diffused fraction of StGBSS–RFP fluorescence (Fig. 4C1, C5–7). The diffuse fluorescence implied that StGBSS might move to the chloroplast stroma from starch granules when coexpressed with StBAM1–GFP (Fig. 4C5), and the protein interaction may lead to the relocalization of StGBSS. Immunoblot analysis demonstrated that each GFP-fusion protein or RFP-fusion protein was intact (Fig. 4D, E), indicating that the fluorescence accurately reflects the localizations of StAmy23, StBAM1, StBAM9, and StGBSS in each case. Therefore, these results provide evidence that StAmy23 is a cytosolic enzyme, StBAM1 is localized in the plastid stroma, and StBAM9 is located on the starch granules. These results may also indicate the subcellular locations where these amylases function.

### *StAmy23*, *StBAM1* and *StBAM9* silencing

To explore the roles of *StAmy23*, *StBAM1*, and *StBAM9* in potato CIS, the expression vectors with *StBAM1* and *StBAM9* silenced individually and collectively were transformed into a CIS-sensitive cultivar, E-potato 3 (E3), using *Agrobacterium*-mediated RNA interference. The vector construction for these RNA interference vectors is shown in Supplementary Fig. S2. To check the specificity of silencing, all of the selected interference fragments were searched using blastn performed in the potato genome database (PGSC *S. tuberosum* group Phureja, DM1-3 Transcripts v3.4). RNAi-*StAmy23* plants from the potato cultivar Solara obtained previously (Ferreira, 2011) were also employed for the functional test. Based on the relative transcript abundance of *StBAM1* and *StBAM9* in all of the transgenic plants (see Supplementary Fig. S3), three transgenic lines exhibiting a lower transcript abundance compared with the untransformed control were selected from each transformation for further function analysis. All of the transgenic lines displayed normal plant morphology and tuber development relative to the corresponding control under normal greenhouse conditions (Supplementary Fig. S4).

**Fig. 5.**
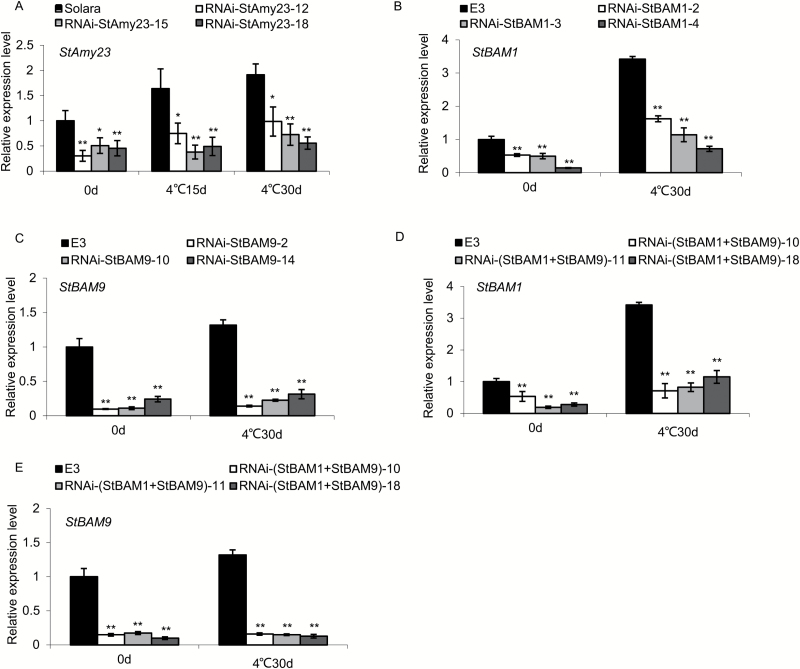
Transcripts of *StAmy23*, *StBAM1*, and *StBAM9* in transgenic tubers stored at 4 °C for 0, 15 and 30 d. (A) The relative expression of *StAmy23* in RNAi-*StAmy23* tubers. (B) The relative expression of *StBAM1* in RNAi-*StBAM1* tubers. (C) The relative expression of *StBAM9* in RNAi-*StBAM9* tubers. (D) The relative expression of *StBAM1* in RNAi-(*StBAM1+StBAM9*) tubers. (E) The relative expression of *StBAM9* in RNAi-(*StBAM1+StBAM9*) tubers. The columns represent the mean values of three biological replicates and the bars indicate the standard deviation. *P < 0.05, **P < 0.01 by Student’s *t* test.

The transgenic tubers were stored at 4 °C for 0, 15 and 30 d to investigate the association between gene transcripts and sugar accumulation. Since both transgenic and untransformed control tubers had little variation in sugar content when stored at 20 °C for up to 30 d (data not shown), the tubers previously stored at 4 °C for 0 day were used for comparison. As shown in Fig. 5, the low temperature generally stimulated gene expression. Compared with the untransformed control, the transcripts of *StAmy23*, *StBAM1*, and *StBAM9* were decreased in the RNAi-lines. For example, *StAmy23* was suppressed by 52–73% in tubers stored at 4 °C for 30 d (Fig. 5A), *StBAM1* by 57–82% (Fig. 5B), and *StBAM9* by 76–89% (Fig. 5C). Obviously, the collective silence of *StBAM1* and *StBAM9* resulted in a dramatic decline in abundance of *StBAM1* and *StBAM9* mRNA showing 66–79% decline for *StBAM1* (Fig. 5D) and 88–91% for *StBAM9* (Fig. 5E). However, transcripts of other *StBAM* genes showed no remarkable decrease (see Supplementary Fig. S5), suggesting a specific silencing of the target genes in the present research.

### StAmy23, StBAM1 and StBAM9 play different roles in potato CIS

**Fig. 6.**
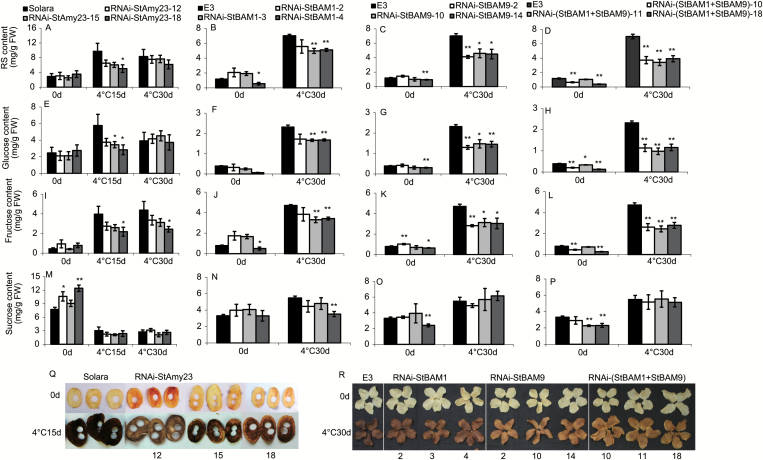
The sugar content and crisp colour of RNAi-*StAmy23*, RNAi-*StBAM1*, RNAi-*StBAM9* and RNAi-(*StBAM1+StBAM9*) tubers stored at 4 °C for 0, 15 and 30 d. (A–D) Reducing sugar (RS) content. (E–H) Glucose content. (I–L) Fructose content. (M–P) Sucrose content. (Q) Colour of potato crisps from RNAi-*StAmy23* tubers stored at 4 °C for 0 and 15 d. (R) Colour of potato crisps from RNAi-*StBAM1*, RNAi-*StBAM9* and RNAi-(*StBAM1+StBAM9*) tubers stored at 4 °C for 0 and 30 d. The columns represent the mean values of three biological replicates and the bars indicate the standard deviation. *P < 0.05, **P < 0.01 by Student’s *t* test.

In addition to RNA transcripts, the contents of RS and sucrose were analysed in the same tubers (Fig. 6). It was obvious that the sucrose content was not affected by the transformation after cold storage (Fig. 6M–P). Looking into individual genes, silencing *StAmy23* did not significantly alter RS, glucose and fructose, although RNAi-*StAmy23*-18 showed a significantly lower fructose content than control in the tubers stored at 4 °C for 30 d (Fig. 6I). These results revealed that StAmy23 might not be the primary factor influencing potato CIS.

In contrast, most of the *StBAM1* (Fig. 6B, F, J) and *StBAM9* (Fig. 6C, G, K) silencing lines accumulated less RS in the tubers stored at 4 °C for 30 d (in total and in glucose and fructose fractions) than in the control (E3). The reduction caused by single gene suppression was enhanced by collective suppression of both *StBAM1* and *StBAM9* (Fig. 6D, H, L; Supplementary Fig. S6). The changes in RS were also reflected by the colour of fried crisps from each of the transgenic tubers. RNAi-*StBAM1* and RNAi-*StBAM9* tubers exhibited visibly lighter crisp colour than the control (E3) after cold storage. A more obvious improvement was observed for RNAi-(*StBAM1+StBAM9*) tubers, while significant differences were not observed for RNAi-*StAmy23* tubers (Fig. 6Q, R). Meanwhile, the crisp colour was visually determined using the Color Standards Reference Chart for Potato Chips. In comparison with the corresponding control, the crisp colour index decreased one to three levels in transgenic tubers (see Supplementary Fig. S7). Together, the results indicate that StBAM1 and StBAM9 are the main contributors to potato CIS. Moreover, we also found that silencing *StBAM9* resulted in a lower RS and lighter crisp colour compared with *StAmy23* or *StBAM1* silenced lines (Supplementary Figs S6 and S7), indicating that StBAM9 may play a more crucial role in potato CIS.

### *StBAM1* and *StBAM9* repression impair starch degradation in potato tubers

**Fig. 7.**
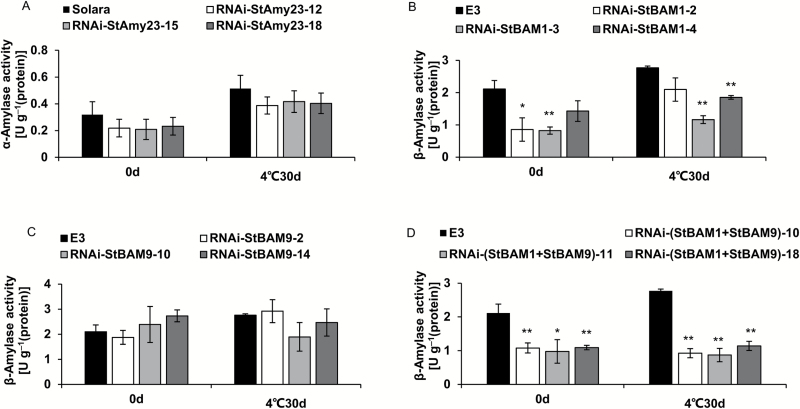
The amylase activity of RNAi-*StAmy23*, RNAi-*StBAM1*, RNAi-*StBAM9* and RNAi-(*StBAM1+StBAM9*) tubers stored at 4 °C for 0 and 30 d. (A) α-Amylase activity of RNAi-*StAmy23* tubers. (B) β-Amylase activity of RNAi-*StBAM1* tubers. (C) β-Amylase activity of RNAi-*StBAM9* tubers. (D) β-Amylase activity of RNAi-(*StBAM1*+*StBAM9*) tubers. The columns represent the mean values of three biological replicates and the bars indicate the standard deviation. *P < 0.05, **P < 0.01 by Student’s *t* test.

To understand how StAmy23, StBAM1, and StBAM9 function in potato CIS, the amylase activities and starch contents of tubers were measured before and after cold storage. The α-amylase activity showed no significant difference in RNAi-*StAmy23* tubers (Fig. 7A), but total β-amylase activity was noticeably reduced in RNAi-*StBAM1* and RNAi-(*StBAM1*+*StBAM9*) tubers and remained unchanged in RNAi-*StBAM9* tubers (Fig. 7B–D). In accordance with structure analysis (Fig. 3), silencing *StBAM9* did not impact the total β-amylase activity, supporting the functional prediction that StBAM9 is an inactive amylase.

**Fig. 8.**
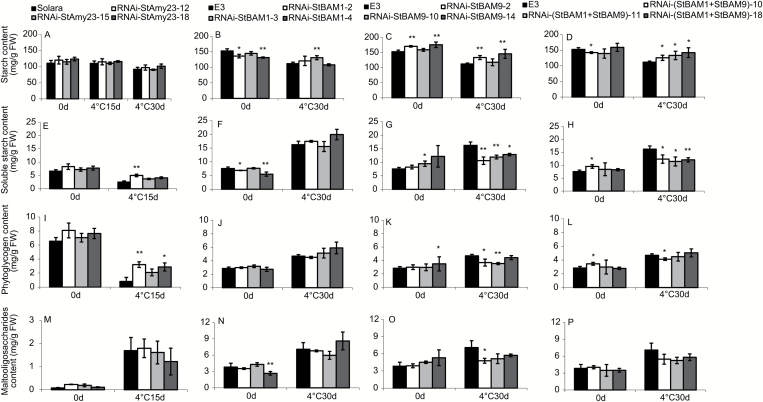
The starch content of RNAi-*StAmy23*, RNAi-*StBAM1*, RNAi-*StBAM9* and RNAi-(*StBAM1+StBAM9*) tubers stored at 4 °C for 0, 15 and 30 d. (A–D) Starch content. (E–H) Soluble starch content. (I–L) Phytoglycogen content. (M–P) Malto-oligosaccharide content. The columns represent the mean values of three biological replicates and the bars indicate the standard deviation. *P < 0.05, **P < 0.01 by Student’s *t* test.

Moreover, compared with the untransformed control, the starch content showed no significant change in RNAi-*StAmy23* tubers (Fig. 8A). However, the starch content of RNAi-*StBAM1* tubers exhibited a remarkable increase in one of the three RNAi lines after cold storage (Fig. 8B). The starch content was elevated in RNAi-*StBAM9* tubers before and after cold storage (Fig. 8C). There was no obvious difference in starch content in RNAi-(*StBAM1+StBAM9*) tubers before cold storage, but it was significantly higher than control (E3) after storage (Fig. 8D), indicating that both StBAM1 and StBAM9 are essential for starch degradation in potato tubers. Furthermore, their synergistic roles may be an underlying mechanism of potato CIS via the starch degradation pathway. Since StBAM9 has no catalytic activity, it may function in a way different from StBAM1.

### StAmy23 and StBAM1 preferentially operate on soluble starch, but StBAM9 acts on starch granules

To clarify the roles of these three amylases in starch breakdown, we measured the soluble starch and its fractions, phytoglycogen and low-molecular-mass malto-oligosaccharide (Fig. 8). Silencing *StAmy23* mainly resulted in an increase in soluble phytoglycogen in cold stored tubers, but there was no obvious change in malto-oligosaccharide (Fig. 8I, M). Although the soluble starch and its fractions showed an increasing trend in *StBAM1*-silenced tubers after cold storage, the increase was not significant (Fig. 8F, J, N). In contrast, the soluble starch content of RNAi-*StBAM9* and RNAi-(*StBAM1*+*StBAM9*) tubers dramatically decreased after cold storage (Fig. 8G, H), and both phytoglycogen and malto-oligosaccharide also declined (Fig. 8K, L, O, P). These observations are in accordance with the subcellular locations of StAmy23, StBAM1, and StBAM9 (Fig. 4), suggesting that they may play distinct roles in starch degradation, particularly under cold conditions. StAmy23 may act on soluble phytoglycogen that is mainly deposited in cytoplasm. StBAM1 may operate on soluble starch accumulated in the amyloplast stroma, while StBAM9, together with other active enzymes, may be involved in the solubilization of starch granules, which might be a prerequisite for the subsequent degradation of the soluble starch by StAmy23, StBAM1 or other enzymes associated with starch degradation.

### StBAM9 and StBAM1 are shown to interact on the starch granules

**Fig. 9.**
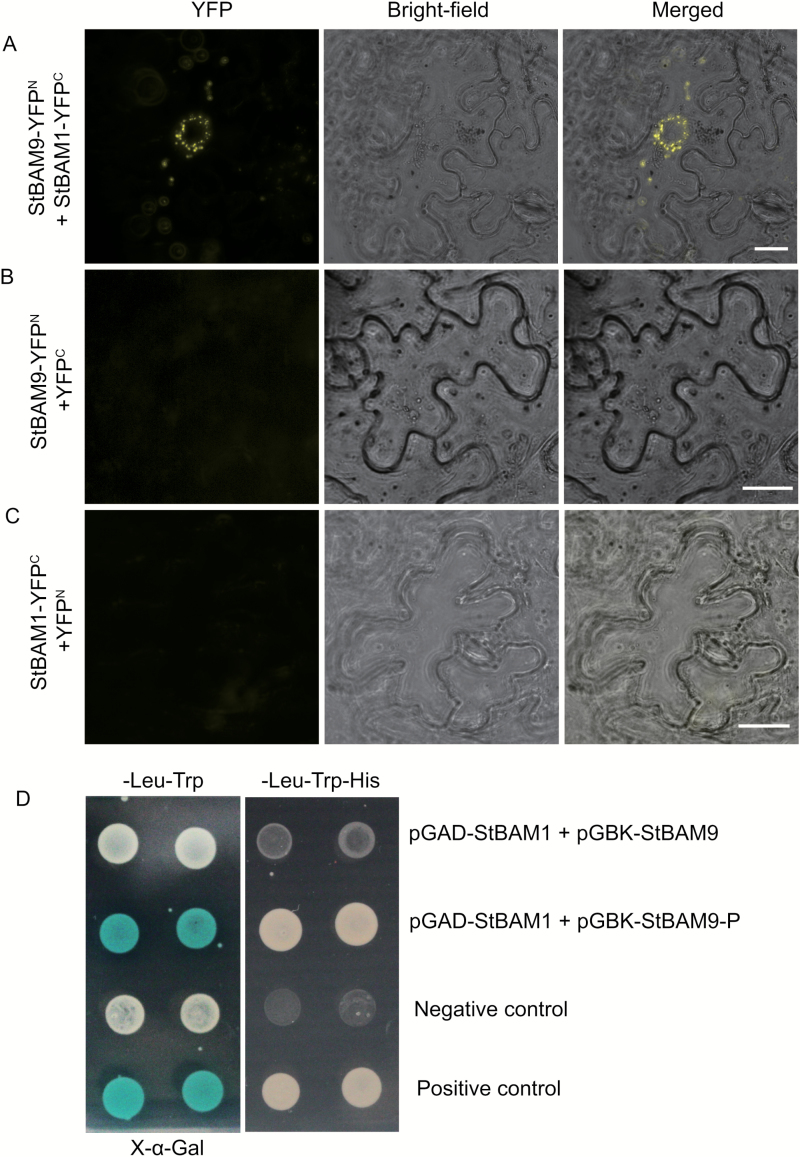
The interaction between StBAM9 and StBAM1. (A–C) StBAM9 and StBAM1 interact on the starch granules in a BiFC assay. (A) Transient coexpression of StBAM9–YFP^N^ and StBAM1–YFP^C^ in *N. benthamiana*. (B) As a negative control, StBAM1–YFP^C^ and YFP^N^ were coexpressed. (C) StBAM9–YFP^N^ and YFP^C^ were coexpressed. Bars: 10 μm. (D) StBAM9 and StBAM1 interact in a yeast two-hybrid assay. Yeast coexpressing StBAM9 and StBAM9-P (StBAM9 without transit peptide) with StBAM1 grows on the double dropout (–Leu–Trp) medium by addition of X-α-Gal and triple dropout (–Leu–Trp–His) medium, respectively. The colour of the clones on SD/–Leu–Trp/X-a-Gal and the survival on SD/–Leu–Trp–His selecting plates represent the interaction.

The interaction between StBAM9 and StBAM1 was accomplished using a bimolecular fluorescence complementation (BiFC) assay in which StBAM9-YFP^N^ (StBAM9 fused with the N-terminal half of yellow fluorescent protein (YFP)) was coexpressed with StBAM1-YFP^C^ (StBAM1 fused with the C-terminal half of YFP) in *N. benthamiana*. We observed the non-uniform distribution and restricted fluorescence pattern with StBAM9–YFP^N^ and StBAM1–YFP^C^ (Fig. 9A), but not with StBAM9–YFP^N^ and YFP^C^ (Fig. 9B), or StBAM1–YFP^C^ and YFP^N^ (Fig. 9C), suggesting that StBAM9 interacts with StBAM1 on the starch granules. The interaction was further confirmed by Gal4-based yeast two-hybrid (Y2H) assay and X-α-Gal assay (Fig. 9D). Interestingly, only StBAM9 without the transit peptide (1–63 aa) interacted with StBAM1, but not full-length StBAM9 in yeast.

## Discussion

The potato genome encodes seven BAM and two AMY proteins relative to nine BAMs and three AMYs in Arabidopsis (Table 1). Consistent with the categories of Arabidopsis, trifoliate orange or apple (Stanley *et al.*, 2002; Fulton *et al.*, 2008; Peng *et al.*, 2014), StBAMs (Fig. 1A) and StAMYs (Fig. 2) were classified into four and three major subfamilies, respectively. This phylogenetic analysis suggests that BAMs and AMYs are evolutionarily conserved in higher plants. Our results demonstrated that potato α-amylase StAmy23 and β-amylases StBAM1 and StBAM9 are involved in the starch degradation in potato CIS. The functional dissection, subcellular positioning and interaction assay led to the conclusion that these amylases have varied impact on potato CIS in different ways.

In general, all of the transgenic tubers at harvest, either by single silencing of *StAmy23*, *StBAM1*, and *StBAM9* or by collective silence of *StBAM1* and *StBAM9*, showed no obvious changes in starch yield per plant and starch granule size quantified by staining with iodine and viewing under a microscope (data not shown). Moreover, no obvious and consistent changes were observed in the content of different kinds of sugars (Fig. 6) and starch (Fig. 8) before cold storage. This result suggested that the observed effects on sugar and starch metabolism in the transgenic tubers after cold storage could be directly attributed to the contribution of StAmy23, StBAM1, and StBAM9.

### StAmy23 may impact on potato CIS by breaking down soluble phytoglycogen in the cytoplasm

Plant AMYs are classified into three major subfamilies, and StAmy23 belongs to subfamily II (Fig. 2) and is localized in the cytoplasm (Fig. 4A), in accordance with the results of Stanley *et al.* (2002). To the best of our knowledge, StAmy23 is the first cytosolic α-amylase experimentally proven *in planta*. The expression of *StAmy23* was strongly induced by low temperature in potato tubers (Zhang *et al.*, 2014*a*). RNAi silencing resulted in the lower accumulation of RS in tubers stored at 4 °C for 15 d and improved crisp colour to some extent (Fig. 6A, E, I, Q), implying that StAmy23 is involved in potato CIS. Interestedly, silencing *StAmy23* yielded a higher accumulation of phytoglycogen in cold stored tubers (Fig. 8I), while the starch degradation was not obviously affected (Fig. 8A), suggesting that StAmy23 might operate on soluble phytoglycogen. Therefore, these results suggest that StAmy23 might impact potato CIS by hydrolysing soluble phytoglycogen, which is a small fraction of potato starch and is mainly deposited in the cytoplasm.

### StBAM1 may play a role in the potato CIS process by hydrolysing soluble starch in the amyloplast stroma

Based on the phylogenetic analysis (Fig. 1A) and protein alignment (Fig. 3), StBAM1 is most closely related to AtBAM1 with the highest sequence similarity, implying they may have a similar function. Previous research showed that *bam1* single mutants showed no changes in starch content in Arabidopsis, but the mutants lacking both *BAM1* and *BAM3* accumulate more starch than the mutant lacking only *BAM3*, suggesting that BAM1 contributes to starch degradation in the absence of BAM3 (Fulton *et al.*, 2008). Similar results were obtained by Monroe *et al.* (2014). Moreover, mutants lacking *BAM1* accumulate more starch in leaf guard cell chloroplasts during the day in Arabidopsis (Valerio *et al.*, 2011).

Our results demonstrated that *StBAM1* repression decreased the total β-amylase activity in cold-stored tubers (Fig. 7B), accompanied by an increase in the starch content (Fig. 8B). As a consequence, the RS content was remarkably reduced (Fig. 6B) and the crisp colour was improved to a certain extent (Fig. 6R). Higher starch accumulation could be a result of a slight accumulation of soluble starch (Fig. 8F), suggesting that StBAM1 may directly break down the soluble starch to regulate potato CIS. This finding is supported by its plastid stromal localization (Fig. 4C) where the soluble starch was deposited. Therefore, it is reasonable to conclude that StBAM1 is the main β-amylase in starch breakdown and plays a major role in potato CIS by hydrolysing soluble starch in the amyloplast stroma.

### StBAM9 plays vital and distinct roles in the starch degradation pathway of potato CIS by acting on starch granules

In the present study, StBAM9 was experimentally confirmed to be located on the starch granules (Fig. 4B). To our knowledge, this is the first report providing evidence for the existence of starch granule-localized β-amylase in potato. Suppressing *StBAM9* resulted in an enhanced starch accumulation (Fig. 8C), together with the apparently decreased RS (Fig. 6C, G, K) and obviously lighter crisp colour (Fig. 6R), the reduction of RS content was more remarkable than silencing *StAmy23* or *StBAM1*. These results indicate a dominant function of StBAM9 in potato CIS. In contrast to suppression of *StAmy23* and *StBAM1*, *StBAM9* repression resulted in a clear decline in soluble starch accumulation (Fig. 8G). Therefore, considering the association of StBAM9 with starch granules, we speculate that StBAM9 might contribute to the release of soluble glucan from the surface of starch granules. All of these findings strongly suggest that unlike StAmy23 or StBAM1, StBAM9 is the primary contributor to potato CIS by directly acting on starch granules.

### StBAM9 may recruit StBAM1 to starch granule and thereby facilitate starch degradation

The gene structure of StBAM9 clustered in the most divergent subfamily IV is different from the other *StBAM* genes (Fig. 1), suggesting that StBAM9 may be a distinct protein in the β-amylase family. Furthermore, StBAM9 is an inactive enzyme (Fig. 3; Zhang *et al.*, 2014*b*). Nevertheless, it is essential in regulating the process of starch metabolism and potato CIS on starch granules. Similarly, AtBAM9 and another β-amylase, AtBAM4, are also inactive, but AtBAM4 plays an important role in starch degradation (Fulton *et al.*, 2008). Thus, this type of protein is proposed to participate in a potentially novel and complex pathway of starch metabolism. The functioning of StBAM9 is worth further investigation. It has been shown that sweet potato β-amylase is a tetramer of identical subunits (Cheong *et al.*, 1995). Isoamylase isoforms ISA1 (catalytic subunits) and ISA2 (non-catalytic subunits) are associated with a multimeric enzyme and form a complex with each other in potato and Arabidopsis (Hussain *et al.*, 2003; Delatte *et al.*, 2005). Moreover, complexes containing class I and class II starch branching enzyme (BE) and complexes containing combinations of starch biosynthetic enzymes SSI, SSII, and class II BE exist in wheat (Tetlow *et al.*, 2004, 2008). Therefore, we speculate that StBAM9 may facilitate starch breakdown by recruiting other proteins to form protein complexes. The paired interactions between amylases (StAmy23, StBAM1, and StBAM9) and StGWD and phosphatases StLSF1, StLSF2, and StGBSS in the Y2H system were investigated (see Supplementary Fig. S8). It identified that only StLSF2 might interact with StBAM9. LSF2 was localized in the chloroplast and specifically hydrolysed the phosphate bound to the C3-position during starch degradation in Arabidopsis (Santelia *et al.*, 2011); complete starch degradation at the starch granule surface requires removal of phosphate (Silver *et al.*, 2014). Therefore, we speculate that the StBAM9–StLSF2 protein complex might bind StLSF2 to the granule surface for further starch degradation. However, we found that the recombinant StBAM9 or StBAM1 had no effect on the dephosphorylation of StLSF2, and StLSF2 also did not affect the activity of StBAM9 or StBAM1 (data not shown). In addition, StBAM1 was captured by StBAM9 from the Y2H library by using StBAM9 without a transit peptide as bait (unpublished data). Moreover, the BiFC and Y2H assay showed that StBAM9 and StBAM1 interacted on the starch granules (Fig. 9); the StBAM9–StBAM1 protein complex may help bind StBAM1 to the starch granule for starch degradation. This is the first report of the existence of an interaction between β-amylases in plants, and provides a new direction for understanding starch metabolism. Furthermore, the activity of recombinant StBAM1 showed no change as the StBAM9 concentration subsequently increased *in vitro* where *p*-nitrophenyl-β-D-maltoheptaoside (the specific substrate for β-amylase) was used as the substrate (data not shown). This result was in accordance with those from the β-amylase activity of silenced *StBAM9* lines (Fig. 7C). Therefore, we speculate that other proteins interacting with StBAM9 may also exist *in vivo*, but further work is needed to test this hypothesis.

In addition, although collective suppression of both *StBAM1* and *StBAM9* resulted in lower RS content than each single repression (Fig. 6D, R), the degree of improvement of CIS was lower than that of the amylase inhibitor *SbAI* overexpression tubers (Zhang *et al.*, 2014*b*). These findings imply that functional redundancy possibly exists, and other amylase family members can compensate for their absence. Moreover, CIS may be regulated by a concerted action of multiple enzymes associated with starch degradation.

In conclusion, potato CIS is regulated by the concerted action of multiple amylases acting on different substrates in different subcellular locations. StBAM9 may release soluble glucan by directly attacking starch granules. The degradation of the soluble glucan was then completed with the participation of the amyloplast stromal StBAM1, cytosolic StAmy23 and other starch degrading enzymes. Moreover, StBAM9 and StBAM1 interact on the starch granules. To the best of our knowledge, this is the first report to demonstrate the interaction between amylases. Moreover, this study provides a novel approach to potato CIS improvement by modulating starch degradation associated with the amylases StAmy23, StBAM1, and StBAM9.

## Supplementary data

Supplementary data are available at *JXB* online.

Fig. S1. Subcellular localizations of free eGFP, RFP and starch granule marker StGBSS in *Nicotiana benthamiana* leaves.

Fig. S2. Constructions of RNA interference vectors.

Fig. S3. The relative expression level of *StBAM1* and *StBAM9* in all of the transgenic potato plants.

Fig. S4. The morphology of RNAi-*StAmy23*, RNAi-*StBAM1*, RNAi-*StBAM9* and RNAi-(*StBAM1+StBAM9*) plants grown in pots.

Fig. S5. The relative expression level of other *StBAM* genes in RNAi-*StBAM1*, RNAi-*StBAM9* and RNAi-(*StBAM1+StBAM9*) tubers.

Fig. S6. The RS content of transgenic tubers containing the different constructs stored at 4 °C for 30 d.

Fig. S7. The crisp colour index of RNAi-*StAmy23*, RNAi-*StBAM1*, RNAi-*StBAM9* and RNAi-(*StBAM1+StBAM9*) tubers after cold storage.

Fig. S8. Interactions between StAmy23, StBAM1, StBAM9 and StLSF2, StLSF1, StGWD, StGBSS proteins.

Table S1. Information for the AMYs and BAMs used to perform the phylogenetic analyses in Figs 1–3.

Table S2. Primers used in this research.

## Supplementary Material

Supplementary_Figures_S1_S8

Supplementary_Tables_S1_S2

## Abbreviations:

AMY: α-amylase
BAM: β-amylase
CIS: cold-induced sweetening;
GWD: glucan water dikinase
RS: reducing sugar.
