# Supplementary material for: Amylases StAmy23, StBAM1 and StBAM9 regulate cold-induced sweetening of potato tubers in distinct ways
Source: J Exp Bot. 2017 Mar 28;68(9):2317–31. doi: 10.1093/jxb/erx076 (PMC5447890; doi:10.1093/jxb/erx076)
Supplement: Supplementary_Figures_S1_S8 [file erx076_suppl_Supplementary_Figures_S1_S8.pdf]

StAmy23, StBAM1 and StBAM9 regulate cold-induced sweetening of potato tubers in distinct ways

Juan Hou, Huiling Zhang, Jun Liu, Stephen Reid, Tengfei Liu, Shijing Xu, Zhendong Tian, Uwe Sonnewald, Botao Song, and Conghua Xie

SUPPLEMENTARY FIGURES

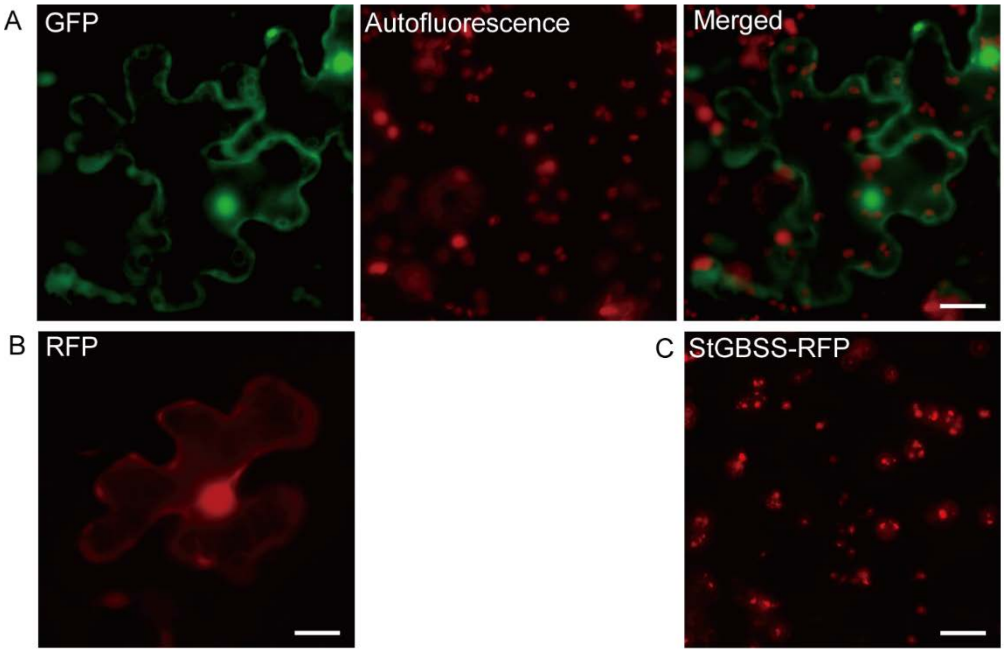

Figure S1. Subcellular localizations of free eGFP, RFP and starch granule marker StGBSS in *Nicotiana benthamiana* leaves. A: The localization of free eGFP. A1: GFP fluorescence; A2: Chlorophyll autofluorescence; A3: Merged image. B: The localization of free RFP. C: The localization of starch granule marker StGBSS-RFP. Bars = 10 μm.

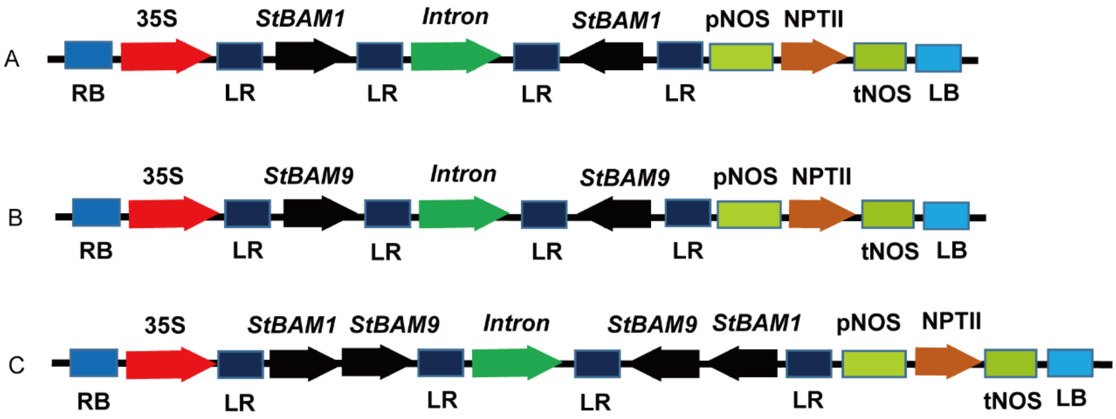

Figure S2. Constructions of RNA interference vectors. A: The construction of RNAi-*StBAM1* vector. B: The construction of RNAi-*StBAM9* vector. C: The construction of RNAi-(*StBAM1*+ *StBAM9*) vector.

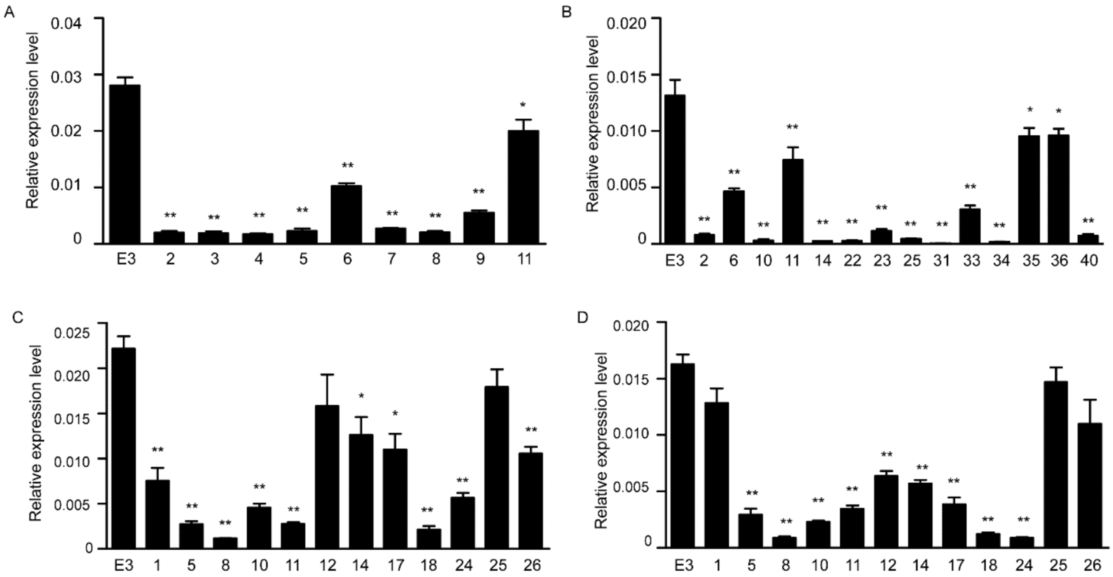

Figure S3. The relative expression level of *StBAM1* and *StBAM9* in all of the potato transgenic plants. A: Transcripts of *StBAM1* in RNAi-*StBAM1* lines. B: Transcripts of *StBAM9* in RNAi-*StBAM9* lines. C: Transcripts of *StBAM1* in RNAi-(*StBAM1*+*StBAM9*) lines. D: Transcripts of *StBAM9* in RNAi-(*StBAM1*+*StBAM9*) lines. Plants were grown under 12 h-light/12 h-dark photoperiod, the leaves for 6 weeks were analysed by quantitative RT-PCR. Three transgenic lines exhibited a lower transcript abundance than the untransformed control (E3) were chosen from each transformation for further function analysis [RNAi-*StBAM1*-2, 3, 4; RNAi-*StBAM9*-2, 10, 14; RNAi-(*StBAM1*+*StBAM9*)-10, 11, 18]. The columns represent the mean values of three biological replicates and the bars indicate the standard deviation of the means. *, $P < 0.05$; **, $P < 0.01$ by Student's *t* test.

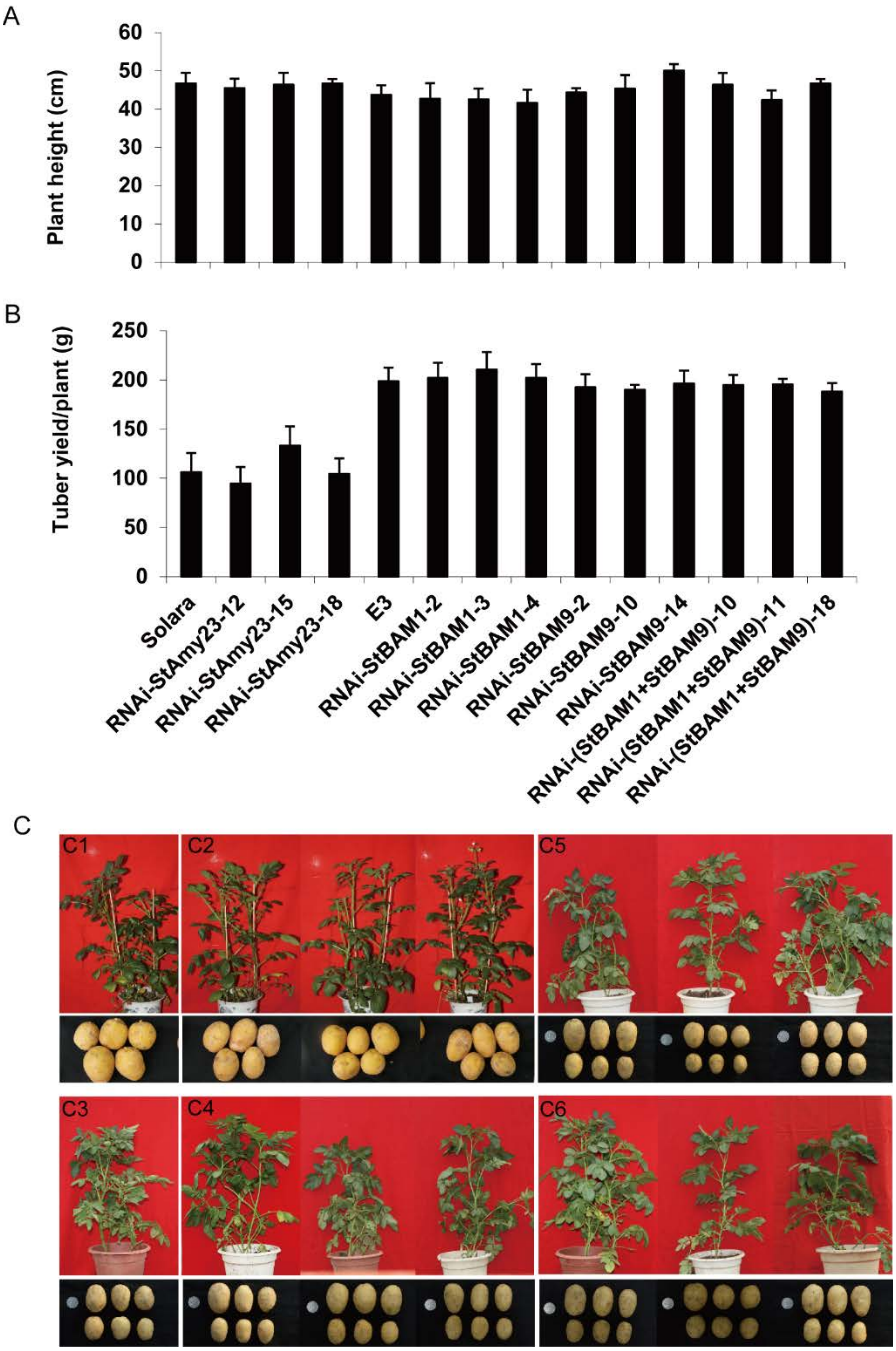

Figure S4. The morphology of RNAi-*StAmy23*, RNAi-*StBAM1*, RNAi-*StBAM9* and RNAi-(*StBAM1*+*StBAM9*) plants grown in pots. A: The plant height of transgenic lines grown for 8 weeks in greenhouse. B: The tuber yield per plant of transgenic lines. C: Plants and tubers of Solara (C1, untransformed control of C2), RNAi-*StAmy23* (C2), E3 (C3, untransformed control of C4-C6)), RNAi-*StBAM1* (C4), RNAi-*StBAM9* (C5) and RNAi-(*StBAM1*+*StBAM9*) (C6). The error bar indicates the standard error of mean of three replicates.

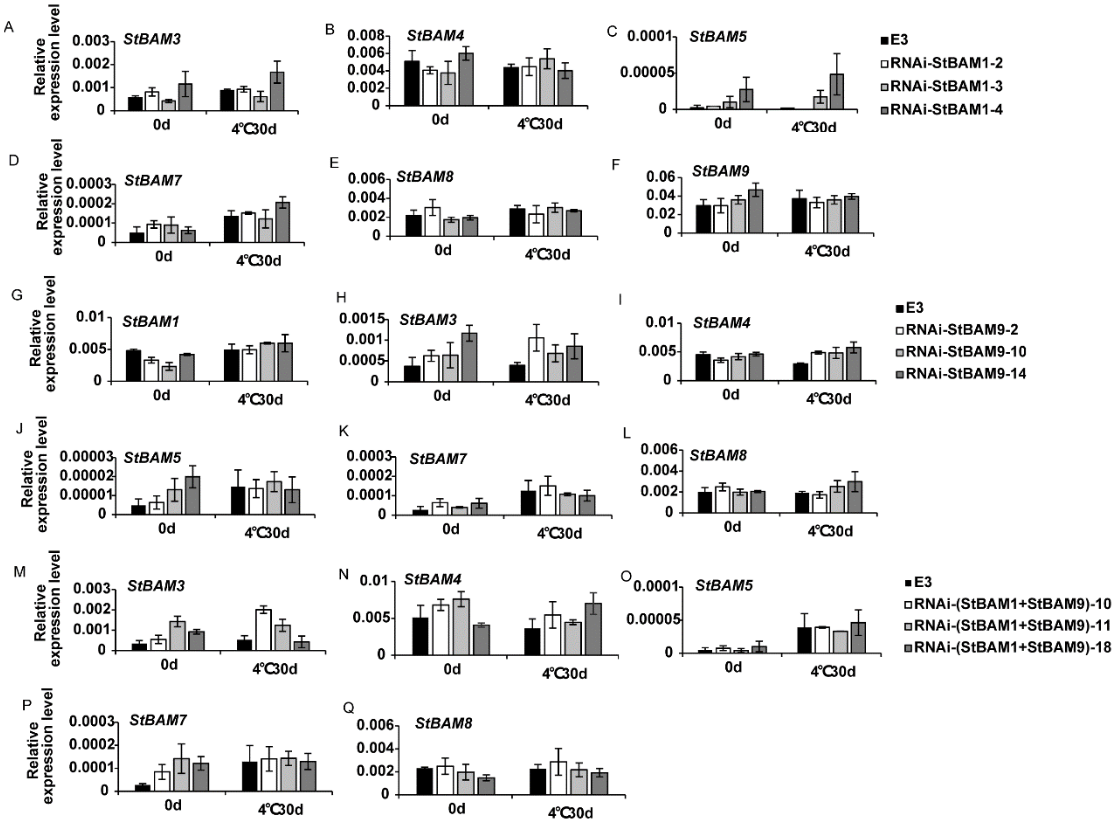

Figure S5. The relative expression level of other *StBAM* genes in RNAi-*StBAM1*, RNAi-*StBAM9* and RNAi-(*StBAM1+StBAM9*) tubers. A-F: Transcripts of other *StBAM* genes in RNAi-*StBAM1* tubers. G-L: Transcripts of other *StBAM* genes in RNAi-*StBAM9* tubers. M-Q: Transcripts of other *StBAM* genes in RNAi-(*StBAM1+StBAM9*) tubers. The columns represent the mean values of three biological replicates and the bars indicate the standard deviation of the means.

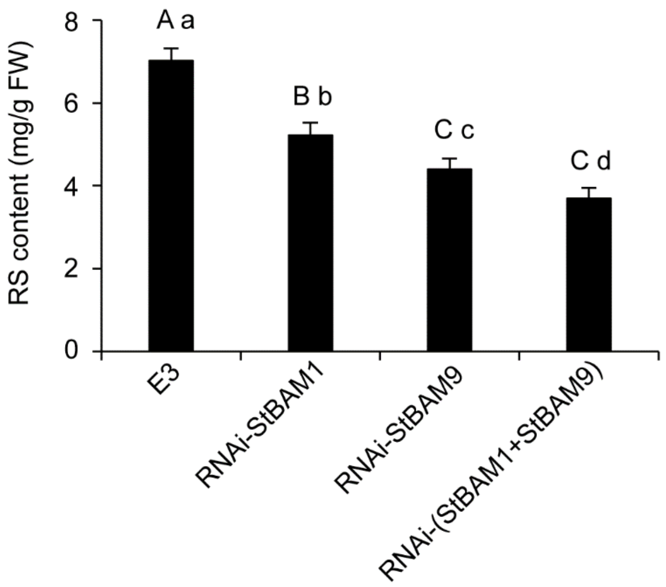

Figure S6. The RS content of transgenic tubers containing the different constructs stored at 4°C for 30 days. The columns represent the mean values of three transgenic lines containing the different constructs, the bars indicate the standard deviation of the means. A LSD test was conducted to compare means and statistically significantly different values are indicated by different letters in the graphs. Capital letters indicate significance at $P = 0.01$, lowercase letters indicate significance at $P = 0.05$.

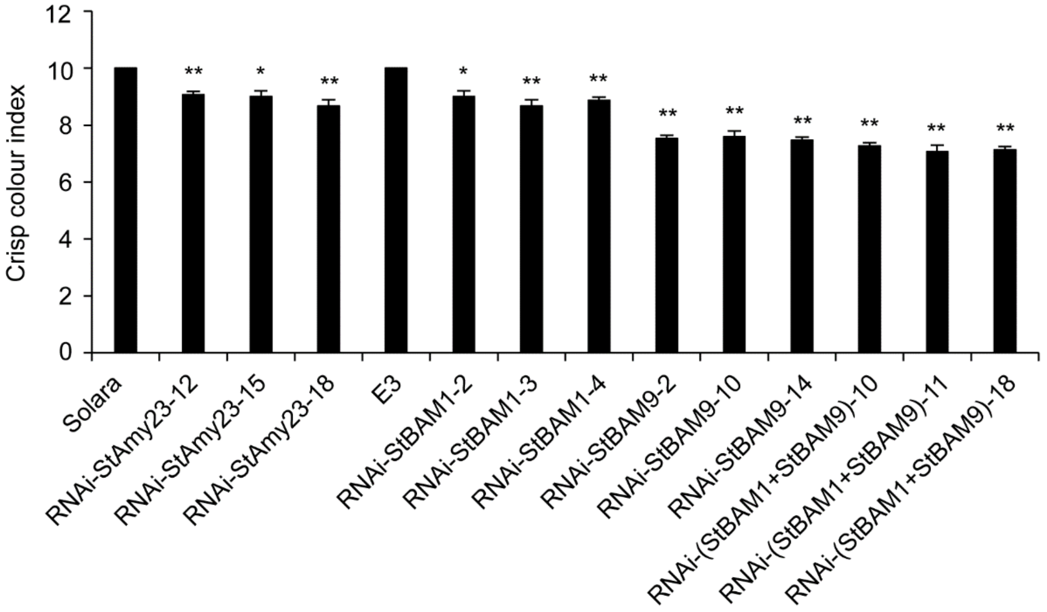

Figure S7. The crisp colour index of RNAi-*StAmy23*, RNAi-*StBAM1*, RNAi-*StBAM9* and RNAi-(*StBAM1*+*StBAM9*) tubers after cold storage. Solara (control) and RNAi-*StAmy23* tubers were stored at 4 °C for 15 days; E3 (control), RNAi-*StBAM1*, RNAi-*StBAM9* and RNAi-(*StBAM1*+*StBAM9*) tubers were stored at 4 °C for 30 days. The crisp colour was visually determined by using the Color Standards Reference Chart for Potato Chips from scale 1 (light) to 10 (dark) (Snack Food Association, USA). The crisp colour index (CCI) was calculated for each line with 15 crisps from three tubers by applying the colour scale value (C) to the formula $CCI = [\sum (C_i \times N_i)] / N$ (where, C_i is colour scale i, N_i is number of crisps of C_i, N is the total number of the crisps tested). The columns represent the mean values and the bars indicate the standard deviation of the means. *, $P < 0.05$; **, $P < 0.01$ by Student's *t* test.

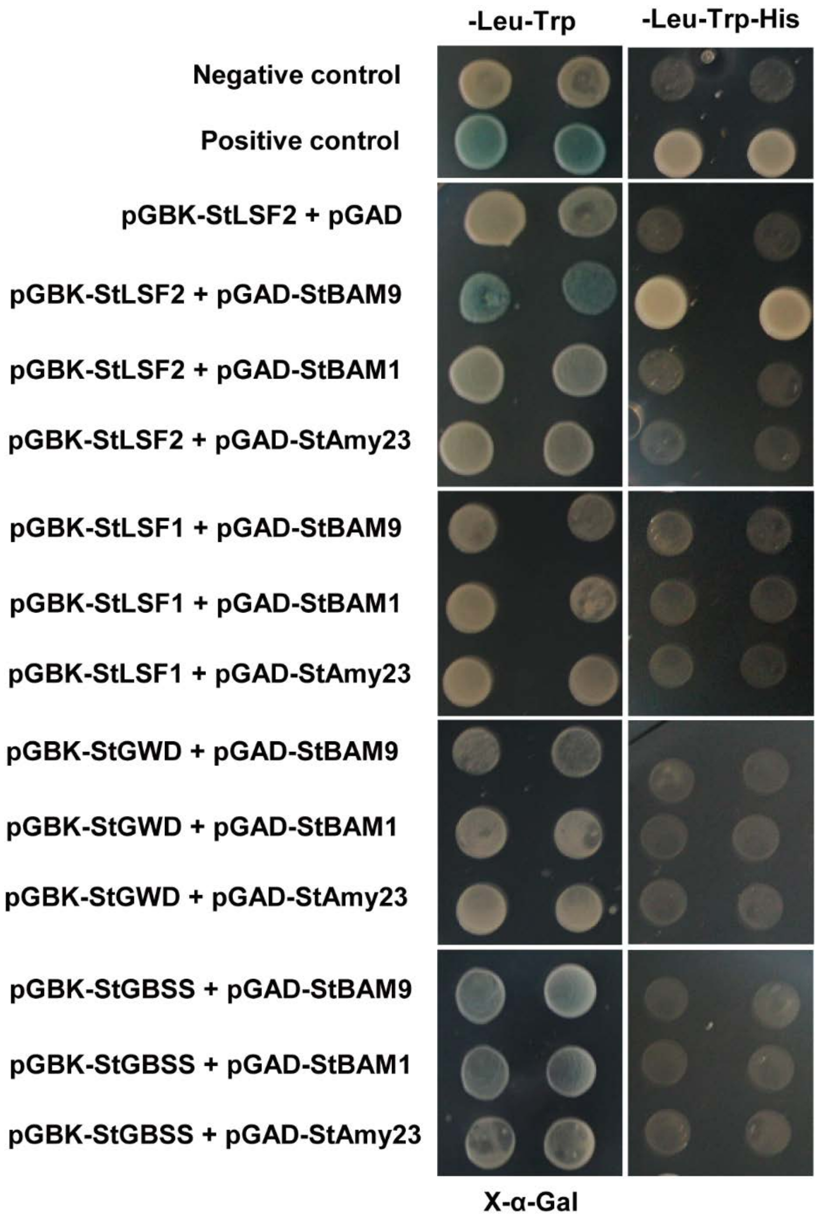

Figure S8. Interactions between StAmy23, StBAM1, StBAM9 and StLSF2, StLSF1, StGWD, StGBSS proteins. Yeast co-expressing StAmy23, StBAM1, StBAM9 with StLSF2, StLSF1, StGWD, StGBSS grows on -Leu-Trp medium by addition of X-α-galactosidase (X-α-Gal) and -Leu-Trp-His medium, respectively. The color of the clones on SD/-Leu-Trp/X-a-Gal and the survival on SD/-Leu-Trp-His selecting plates represent the interaction between StBAM9 and StLSF2 assayed with the BD Matchmaker Screening Kit (Clontech). The positive and negative controls are represented by pGBK-53/pGADRecT[b] and pGBK-Lam/pGAD-RecT[b], respectively.
