# Supplementary material for: Amylases StAmy23, StBAM1 and StBAM9 regulate cold-induced sweetening of potato tubers in distinct ways
Source: J Exp Bot. 2017 Mar 28;68(9):2317–31. doi: 10.1093/jxb/erx076 (PMC5447890; doi:10.1093/jxb/erx076)
Supplement: Supplementary_Tables_S1_S2 [file erx076_suppl_Supplementary_Tables_S1_S2.pdf]

StAmy23, StBAM1 and StBAM9 regulate cold-induced sweetening of potato tubers in distinct ways

Juan Hou, Huiling Zhang, Jun Liu, Stephen Reid, Tengfei Liu, Shijing Xu, Zhendong Tian, Uwe Sonnewald, Botao Song, and Conghua Xie

Supplementary Table S1. Information for the AMYs and BAMs used to perform the phylogenetic analyses in Figure 1-3.
Potato Genome Sequence Consortium (PGSC) database names of BAMs and AMYs in potato, GenBank accession numbers in Arabidopsis, barley, soybean and Poncirus, Locus names of tomato, rice and poplar, UniProt database names of apple are provided, respectively.

Name	PGSC name	GenBank accession	Locus name	UniProtKB name
Solanum tuberosum BAM1	PGSC0003DMP400002800			
Solanum tuberosum BAM3	PGSC0003DMP400035625			
Solanum tuberosum BAM4	PGSC0003DMP400021443			
Solanum tuberosum BAM5	PGSC0003DMP400045472			
Solanum tuberosum BAM7	PGSC0003DMP400000368			
Solanum tuberosum BAM8	PGSC0003DMP400041754			
Solanum tuberosum BAM9	PGSC0003DMP400018848			
Solanum lycopersicum 1			Solyc09g091030	
Solanum lycopersicum 2			Solyc01g094580	
Solanum lycopersicum 3			Solyc07g052690	
Solanum lycopersicum 4			Solyc08g005780	
Solanum lycopersicum 5			Solyc08g007130	
Solanum lycopersicum 6			Solyc08g077530	
Solanum lycopersicum 7			Solyc08g082810	
Arabidopsis thaliana BAM1		NP189034		
Arabidopsis thaliana BAM2		CAB80858		
Arabidopsis thaliana BAM3		CAB58423		
Arabidopsis thaliana BAM4		AAK76508		
Arabidopsis thaliana BAM5		CAB78563		
Arabidopsis thaliana BAM6		AAM97128		
Arabidopsis thaliana BAM7		AAC28536		

Arabidopsis thaliana BAM8	BAB10251	
Arabidopsis thaliana BAM9	AAL47434	
Oryza sativa 1		Os01g13550
Oryza sativa 2		Os02g03690
Oryza sativa 3		Os03g04770
Oryza sativa 4		Os03g22790
Oryza sativa 5		Os07g35880
Oryza sativa 6		Os07g35940
Oryza sativa 7		Os07g47120
Oryza sativa 8		Os09g39570
Oryza sativa 9		Os10g32810
Oryza sativa 10		Os10g41550
Hordeum vulgare Bmy1	CAC16789	
Hordeum vulgare Bmy2	AAX37357	
Glycine max GmBMY1	P10538	
Glycine max Bmy1	CAI39245	
Glycine max Bmy2	CAI39244	
Poncirus trifoliata BAM1	JX139692	
Poncirus trifoliata BAM2	JX139693	
Poncirus trifoliata BAM3	JX139694	
Poncirus trifoliata BAM4	JX139695	
Poncirus trifoliata BAM5	JX139696	
Poncirus trifoliata BAM6	JX139697	
Poncirus trifoliata BAM7	JX139698	
Poncirus trifoliata BAM8	JX139699	
Populus trichocarpa 1		POPTR_0017s06840g
Populus trichocarpa 2		POPTR_0003s08360g
Populus trichocarpa 3		POPTR_0003s03330g
Populus trichocarpa 4		POPTR_0001s11000g

Populus trichocarpa 5			POPTR_0003s14340g	
Populus trichocarpa 6			POPTR_0008s20870g	
Populus trichocarpa 7			POPTR_0014s07950g	
Populus trichocarpa 8			POPTR_0008s17420g	
Populus trichocarpa 9			POPTR_0010s07340g	
Populus trichocarpa 10			POPTR_0003s10570g	
Solanum tuberosum Amy1	PGSC0003DMP400035807			
Solanum tuberosum Amy23	PGSC0003DMP400017447			
Arabidopsis thaliana AMY1		At1g69830		
Arabidopsis thaliana AMY2		At1g76130		
Arabidopsis thaliana AMY3		At4g25000		
Hordeum vulgare AMY1		P00693		
Hordeum vulgare AMY2		P04063		
Hordeum vulgare AMY3		P04747		
Hordeum vulgare AMY4		P04748		
Hordeum vulgare AMY5		P04749		
Hordeum vulgare AMY6		P04750		
Malus domestica AMY2				Q5BLY2
Malus domestica AMY8				Q9M6R9
Malus domestica AMY9				Q5BLY0
Malus domestica AMY10				Q5BLY3

Supplementary Table S2. Primers used in this research.

Primer	Sequence(5'-3')	Related experiment
StAmy23-F	ATGGGGCTTGATGAAAGTCA	Gene cloning
StAmy23-R	TTACTTCTGCCAGACTGCAT	Gene cloning
StBAM1-F	ATGGCGATGAGTATGCCACA	Gene cloning
StBAM1-R	TTAGTGCATGAGGGCCATTGC	Gene cloning
StBAM9-F	ATGGAGGTTTCAGTGATGGG	Gene cloning
StBAM9-R	CTAAGCTGCTTGCATCTGGA	Gene cloning
StAmy23-GFP-F	AAAAAGCAGGCTCAATGGGGCTTGATGAAAGTCA	Subcellular localization
StAmy23-GFP-R	AGAAAGCTGGGTACTTCTGCCAGACTGCATAGC	Subcellular localization
StBAM1-GFP-F	AAAAAGCAGGCTCAATGGCGATGAGTATGCCACAC	Subcellular localization
StBAM1-GFP-R	AGAAAGCTGGGTAGTGCATGAGGGCCATTGC	Subcellular localization
StBAM9-GFP-F	AAAAAGCAGGCTCAATGGAGGTTTCAGTGATGGGAA	Subcellular localization
StBAM9-GFP-R	AGAAAGCTGGGTAAGCTGCTTGCATCTGGAGATGA	Subcellular localization
StGBSS-RFP-F	AAGCTGACTCTAGCAGATCTATGGCAAGCATCACAGCTTC	Subcellular localization
StGBSS-RFP-R	ATGCTAGAATCGATAGATCTGGGAGTGGCTACATTTTCCT	Subcellular localization
StBAM1-C1254	AAAAAGCAGGCTATACTACAACACCCGTAACCGAGAT	RNAi
StBAM1-C1626	AGAAAGCTGGGTCATTTTCTTCACAAAGGCAACG	RNAi
StBAM9-T1620	AAAAAGCAGGCTTCTCCTGAACATTTCCCTGC	RNAi
StBAM9-T1900	AGAAAGCTGGGTAAGCCAACAAACTTTCTCATACTAT	RNAi
(StBAM1+StBAM9)-1-C751	AAAAAGCAGGCTGTCCAATGCCATTCTGATTTC	RNAi
(StBAM1+StBAM9)-1-C989	TAAGTCGTCTCCCAAGATCCCCCCATTCAGGCTTACCAA	RNAi
(StBAM1+StBAM9)-9-C976	GGTAAGCCTGAATGGGGGGATCTTGGGAGACGACTTATG	RNAi
(StBAM1+StBAM9)-9-C1193	AGAAAGCTGGGTCCATCTCTGTTGGCTGTGTTAT	RNAi
BK-StBAM1-F	ATGGCCATGGAGGCCGAATTCATGGCGATGAGTATGCCACA	Y2H
BK-StBAM1-R	ATGCGGCCGCTGCAGGTCGACTTAGTGCATGAGGGCCATTG	Y2H
BK-StBAM9-F	ATGGCCATGGAGGCCGAATTCATGGAGGTTTCAGTGATGGG	Y2H
BK-StBAM9-R	ATGCGGCCGCTGCAGGTCGACCTAAGCTGCTTGCATCTGGA	Y2H

BK-StBAM9-P-F	ATGGCCATGGAGGCCGAATTCATGGCATCTTCTTGTTCACAAC	Y2H
BK-StBAM9-P-R	ATGCGGCCGCTGCAGGTCGACCTAAGCTGCTTGCATCTGGA	Y2H
BK-StGBSS-F	ATGGCCATGGAGGCCGAATTCATGGCAAGCATCACAGCTTC	Y2H
BK-StGBSS-R	ATGCGGCCGCTGCAGGTCGACTTAGGGAGTGGCTACATTTT	Y2H
BK-StGWD-F	ATGGCCATGGAGGCCGAATTCATGGATTCTATGCATCTGTC	Y2H
BK-StGWD-R	ATGCGGCCGCTGCAGGTCGACTCACTGGGGTTGAGGTCGCG	Y2H
BK-StLSF1-F	ATGGCCATGGAGGCCGAATTCATGTGTTCACTACAACTTCC	Y2H
BK-StLSF1-R	ATGCGGCCGCTGCAGGTCGACCTATTTAGGTGCTAGCGTTA	Y2H
BK-StLSF2-F	ATGGCCATGGAGGCCGAATTCATGGAGGAATACAACTTAGC	Y2H
BK-StLSF2-R	ATGCGGCCGCTGCAGGTCGACTCAAGTGTCACGAAGGGCAC	Y2H
StBAM1-YFP^{C}-F	CTGTTGATACATATGGGATCCATGGCGATGAGTATGCCACA	BiFC
StBAM1-YFP^{C}-R	ACCGAATTCACTAGTGTCGACGTGCATGAGGGCCATTGC	BiFC
StBAM9-YFP^{N}-F	TTGATACATATGGGATCCATGGAGGTTTCAGTGATGGG	BiFC
StBAM9-YFP^{N}-R	GAATTCACTAGTGTCGACAGCTGCTTGCATCTGGAGAT	BiFC
